# Protein Lactylation in Cancer: Mechanisms and Therapeutic Targets

**DOI:** 10.1002/mco2.70675

**Published:** 2026-03-10

**Authors:** Qianying Ouyang, Qianyu Hu, Caiqin Wang, Yizi He, Ruolan Zeng, Yajun Li, Chang Su, Guige Lu, Xueting Zhu, Ling Xiao, Hui Zhou

**Affiliations:** ^1^ Department of Lymphoma & Hematology The Affiliated Cancer Hospital of Xiangya School of Medicine Central South University/Hunan Cancer Hospital Changsha China; ^2^ Department of Histology and Embryology Xiangya School of Basic Medical Sciences Central South University Changsha China; ^3^ Graduate Collaborative Training Base of Hunan Cancer Hospital University of South China Hengyang China

**Keywords:** lactate, lactylation, metabolic‐epigenetic axis, precision oncology

## Abstract

The Warburg effect states that cancer cells preferentially undergo aerobic glycolysis, producing lactate as a key metabolic byproduct. Lactate acidifies the tumor microenvironment (TME) and serves as a signaling molecule and substrate for lysine lactylation (Kla), a novel posttranslational modification (PTM) discovered in 2019 that links glycolytic metabolism to epigenetic and proteomic reprogramming. The reversible modification of histones and nonhistone proteins orchestrates oncogenic adaptation and drives tumor progression. However, gaps persist in our understanding of the multifactorial regulation of lactylation and its translational potential in overcoming tumor heterogeneity and resistance. This review highlights the emerging roles of lactylation in cancer therapies, including the enhancement of DNA repair mechanisms during chemotherapy, stabilization of key signaling effectors upon targeted therapy, and promotion of an immunosuppressive TME in immunotherapy. We further examined regulatory factors associated with lactylation, from competitive PTMs and genetic mutations to microbial influences and environmental signals. Additionally, we discuss the therapeutic potential of targeting lactylation via indirect modulators currently under investigation and the visualization of lactate and lactylation modifications. By synthesizing these insights, this review highlights lactylation as a reversible metabolic‐epigenetic axis for precision oncology, enabling predictive biomarkers, combination strategies, and novel interventions to address the dynamic challenges of cancer.

## Graphical abstract

This schematic illustrates the central role of lactylation, a lactate‐derived posttranslational modification, in linking metabolic reprogramming to cancer progression and therapy resistance. At its core, lactylation modulates proteins, influenced by metabolic shifts and environmental factors. The key pathways involved in this process include glucose metabolism via the tricarboxylic acid (TCA) cycle, producing lactate that drives protein lactylation and lowers the extracellular pH, generating an acidic tumor microenvironment (TME). Regarding drug response, lactylation affects chemotherapy response by regulating DNA modification as well as tumor proliferation and apoptosis (arrows represent promotion and termination lines represent inhibition). The lactylation of functional proteins and histones may influence the mechanism of targeted drug action, as lactylation modification of the target protein itself could potentially lead to resistance or loss of efficacy of targeted therapies. Lactylation can also weaken the therapeutic efficacy of CAR‐T cell therapy and other immunotherapies, and may also induce an inhibitory TME. Regarding factors affecting lactylation, competing modifications like acetylation (Ac), ubiquitination (Ub), and phosphorylation (P) integrate with lactylation. External factors encompass genetic mutations, signaling pathways, the gut microbiome, drugs, viruses, and pollutants like PM2.5; all of these reprogram metabolism. Overall, lactylation, as a metabolic‐epigenetic nexus, serves as a target for enhancing cancer therapies by disrupting lactate‐driven metabolic reprogramming.

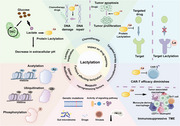

## 1 Introduction

The metabolic reprogramming of cancer cells, as explained by the Warburg effect, which was first described by Otto Warburg in 1924, has long been recognized as a hallmark of malignancy. Tumors preferentially utilize aerobic glycolysis to produce lactate, even in the presence of oxygen, thereby supporting rapid proliferation and survival under stress [1, 2, 3]. Historically viewed as a metabolic waste product, lactate has evolved into a multifaceted molecule serving as an energy source, signaling mediator, and modulator of the tumor microenvironment (TME), influencing processes such as angiogenesis, immune evasion, and extracellular acidification [4, 5, 6]. A pivotal advancement occurred in 2019, when Zhang et al. identified lysine lactylation (Kla) as a novel posttranslational modification (PTM), linking glycolytic metabolism directly to epigenetic regulation through the modification of histones and nonhistone proteins [7]. This discovery bridged the cellular metabolism with gene and protein functions. By 2025, studies had expanded to include diverse lactylation isomers (e.g., L‐, D‐, and S‐lactylation) and their roles in cancer [8, 9, 10].

Current research on lactylation in cancer has revealed its profound implications in tumor progression, heterogeneity, and therapeutic resistance. For instance, histone lactylation at certain sites, such as H3K18la and H4K12 lactylation (H4K12la), has been linked to enhanced autophagy, ferroptosis resistance, and immune suppression, contributing to poor prognosis and therapy evasion. Recent advances have also underscored the role of lactylation in nonhistone proteins in non‐small cell lung cancer (NSCLC), breast cancer, and colon cancer, such as MRE11 and NBS1, which enhance DNA damage repair and chemoresistance [11, 12, 13]. Moreover, emerging evidence has highlighted the involvement of lactylation in immune checkpoint regulation and TME remodeling, positioning it as a critical node in immunotherapy resistance. Despite these insights, the field remains fragmented, with gaps in our understanding of the multifactorial influences on lactylation and its translational potential still remaining.

This review is motivated by the urgent need to synthesize the rapidly evolving landscape of lactate and lactylation research amid the challenges of tumor heterogeneity and dynamic metabolism, which complicate effective cancer therapies. With lactate emerging as a reversible metabolic‐epigenetic bridge, it offers novel avenues for overcoming resistance to conventional treatments. By consolidating recent findings, this article aims to highlight the therapeutic modulation potential of lactate and lactylation, emphasizing breakthroughs such as the targeted inhibition of lactylation to sensitize tumors to drugs and their biomarker utility for personalized medicine.

In this review, we explore the implications of metabolic lactate in tumors, including its impact on intratumoral pH and the promotion of protein lactylation. Subsequently, we investigated the interplay between tumor lactate, lactylation, and drug treatment, covering their roles in chemotherapy resistance via DNA repair and survival pathways, targeted drug efficacy through oncogenic signaling and autophagy, and immunotherapy by shaping the immunosuppressive microenvironment. We then summarized the factors influencing lactylation‐modulated therapy, including other PTMs and metabolic reprogramming driven by genetic mutations, signaling pathways, body flora, and other elements. Finally, we discuss the clinical trials and visualization of lactylation‐related drugs and conclude with prospects for their application in precision oncology.

## 2 Roles of Lactate in Tumors

**FIGURE 1:**
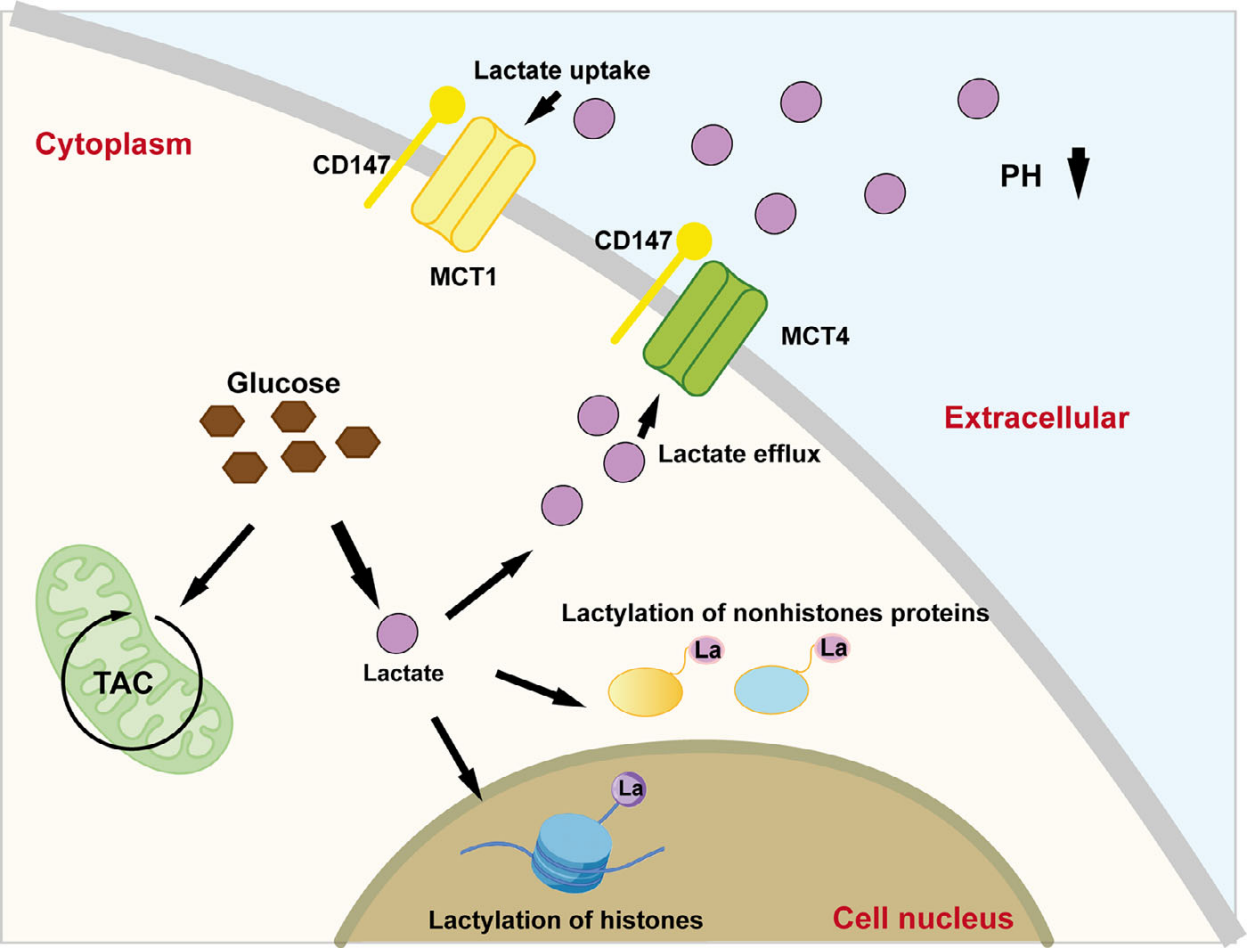
The generation and destination of lactate. Tumor cells produce lactate via glycolytic metabolism. Lactate can translocate into the nucleus to mediate histone lactylation or modify nonhistone proteins in the cytoplasm. Furthermore, lactate is transported across the plasma membrane by monocarboxylate transporters (MCTs), predominantly MCT4 for efflux and MCT1 for influx, and its extracellular accumulation contributes to acidification of the tumor microenvironment through pH reduction.

Lactate, a key metabolic byproduct of cancer cells, plays multifaceted roles in tumor progression by altering the intratumoral microenvironment and influencing epigenetic and cellular processes. Primarily generated through the Warburg effect, metabolic reprogramming that favors aerobic glycolysis over oxidative phosphorylation‐lactate accumulation not only acidifies the TME but also serves as a substrate for PTMs that drive oncogenic adaptations (Figure 1). This section explores the contribution of lactate to intratumoral pH regulation and the promotion of protein lactylation, highlighting its implications in cancer metabolism, proliferation, and therapeutic resistance.

### 2.1 Lactate Promotes Intratumoral pH Changes

Under hypoxic conditions, pyruvate is converted to lactate by lactate dehydrogenase (LDH) [1]. However, in cancer cells, this conversion occurs even with sufficient oxygen, through a process known as aerobic glycolysis [14, 15, 16]. Glycolysis is an inefficient energy pathway, producing only two ATP molecules per glucose molecule compared to 36–38 ATP molecules from oxidative phosphorylation, prompting tumors to consume more glucose than normal tissues and generate abundant lactate. This metabolic shift not only supports rapid cell proliferation, but also confers advantages such as resistance to apoptosis and enhanced biosynthetic capabilities [4, 17, 18].

Lactate is produced intracellularly and rapidly exported to the extracellular space via monocarboxylate transporters (MCTs), a family of proton‐linked transporters encoded by the *SLC16* gene family, which facilitate the production of lactate and H^+^ ions across the plasma membrane [5]. MCTs are critical for maintaining intracellular pH homeostasis and extracellular acidification in tumors, with at least 14 isoforms identified, although MCT1 and MCT4 are the most prominent in the cancer contexts [19]. MCT1 exhibits a high affinity for lactate and primarily mediates lactate uptake in oxidative cells, enabling metabolic symbiosis, where lactate from glycolytic cancer cells is recycled as fuel [20, 21]. In contrast, MCT4 has a low affinity to lactate and high capacity, predominantly driving lactate efflux from highly glycolytic cancer cells to prevent intracellular acidification and support the Warburg phenotype. MCT2 exhibits the highest lactate affinity to facilitate uptake. MCT3 with an affinity similar to MCT1, mainly aids lactate efflux in glycolytic contexts for pH regulation, particularly in central nervous system tumors such as gliomas, but its roles in broader oncogenesis are less characterized and thus underexplored [22]. This bidirectional transport is tightly regulated by factors such as HIF‐1α, which upregulates MCT4 under hypoxia, and CD147/basigin, a chaperone essential for the surface expression of MCT1/4 [22, 23]. The efflux establishes a mildly acidic TME, typically with a pH ranging from 6.5 to 6.9, compared with the neutral pH (approximately 7.4) in normal tissues [24]. The acidification of the TME hinders the clearance of acidic metabolites and lactate shuttles between cells via MCTs, further promoting cancer progression. This acidity fosters neoplastic growth by enhancing cancer cell proliferation, migration, invasion, and metastasis through mechanisms such as extracellular matrix (ECM) degradation by proteases, increased angiogenesis via the upregulation of vascular endothelial growth factor, and resistance to apoptosis in tumor cells, while being toxic to the surrounding normal cells [25]. Furthermore, the acidic TME contributes to immune evasion by impairing immune cell function, such as by inhibiting T cell activation and promoting immunosuppressive phenotypes in macrophages and myeloid‐derived suppressor cells, thereby driving tumor escape from immunosurveillance [26]. In terms of drug responses, acidity exacerbates therapeutic resistance by altering drug ionization and uptake (e.g., reducing the efficacy of weakly basic chemotherapeutics, such as doxorubicin), promoting multidrug resistance through efflux pumps, and inducing adaptive cellular changes under hypoxic and acidic stress [27]. Strategies to alkalize the TME, such as the use of bicarbonate buffers or proton pump inhibitors, have the potential to reverse these effects and improve treatment outcomes in preclinical models [28, 29]. Specific cancers such as pancreatic and gastric tumors are particularly sensitive to acidic conditions that accelerate invasion and metastasis. Beyond pH modulation, lactate also acts as a signaling molecule, activating pathways like HIF‐1α stabilization, which enhances angiogenesis and metabolic adaptation [19].

### 2.2 Lactate Promotes Protein Lactylation

In 2019, lactylation was identified as a novel PTM in which lactate‐derived lactyl‐CoA modifies lysine residues on histones, linking metabolism to epigenetics [7]. H3K18la is the most studied site of histone lysine lactylation, regulating gene expression, intracellular signaling, and protein function to drive cancer proliferation, migration, and invasion [7, 30, 31].

In addition to histones, lactylation extends to nonhistone proteins, broadening their impact on tumor progression [7, 11, 30]. For instance, in cervical cancer, high lactate levels induce DCBLD1 lactylation, stabilize DCBLD1 expression, and activate the pentose phosphate pathway via glucose‐6‐phosphate dehydrogenase (G6PD) upregulation, fueling nucleotide synthesis and tumor growth [32]. In colorectal cancer (CRC), METTL3 enhances m6A RNA modifications, promotes immunosuppression in tumor‐infiltrating myeloid cells (TIMs). Nonhistone targets such as MRE11 (K673la) enhance DNA repair and chemoresistance, whereas NBS1 lactylation supports homologous recombination.

Recent advances have revealed the role of lactylation in drug resistance, such as in bevacizumab‐resistant CRC, via H3K18la‐mediated autophagy. In immunotherapy, lactylation reprograms macrophages toward immunosuppressive phenotypes, thereby reducing CAR‐T cell efficacy. Pharmacological targeting of lactylation, for example, via LDH inhibitors or delactylases, has emerged as a strategy to overcome resistance and inhibit progression, with ongoing studies highlighting its biomarker potential in personalized therapy.

Similar to other PTMs of proteins, lactylation is now widely recognized to be mediated by “writers,” “erasers,” and “readers,” among other factors. Lactylation occurs when multiple lactate pathways condense with intracellular coenzyme A to produce lacCoA. Then, in the presence of an acyltransferase known as a “writer,” lac‐CoA transfers the lactate acyl group to the lysine site. The “eraser” recognizes the lactide acyl group and unbinds it from the lysine site. The “reader” then recognizes the information and leads to changes in gene expression and protein function, ultimately altering the cellular phenotype. The “writers” verified in in vitro and in vivo experiments include P300 [33], AARS 1 [34], and HBO1 [35], while the de‐lactylating “erasers” are mainly HDAC1‐3 and SIRT1‐3 [36]. The catalytic mechanism and physiological function of histone deacetylases (HDACs) and sirtuins are not identical. Compared to other sirtuins, SIRT3 is more active against the H4K16la site [37]. The identified readers include DPF2, which binds to H3K14la via its double PHD finger domain with high affinity. This facilitates chromatin recruitment and the activation of oncogenes (e.g., *SEMA5A* and *ROCK1*) and promotes cell proliferation and survival in lactate‐enriched environments, particularly driving tumorigenesis in cervical cancer through metabolic‐epigenetic linkages [38]. Another reader is Brg1, which interacts with H3K18la through its bromodomain, enabling chromatin remodeling. This enhances the accessibility and transcription of genes involved in mesenchymal‐epithelial transition (e.g., *Cdh1* and *Epcam*) and pluripotency (e.g., *Oct4* and *Nanog*) during early iPSC reprogramming, thereby linking metabolic shifts such as glycolysis to epigenetic regulation [33]. Some studies have shown that targeting the activity of writer enzymes in vitro and in vivo can influence the course of the disease by altering the level of lactylation modifications [39].

## 3 Lactylation and Its Effects on Anticancer Treatment

The discovery of lactylation as a critical regulatory mechanism in various essential biological processes and its profound influence on the fate of tumor cells has led to a growing body of research exploring its role in the efficacy of both traditional and innovative drug therapies, including chemotherapy, targeted therapies, and immunotherapy (Table 1).

**TABLE 1:** Lactylation targets of tumor therapies.

Drug classification	Drug	Protein	Site	Function	Drug response	Source
Chemotherapy	Cisplatin	H3	K18	Activate the expression of YBX1 and YY1	Resistance	[50]
	Cisplatin, etc.	MRE11	K673	Promote DNA terminal excision and homologous recombination repair	Resistance	[11]
	Irinotecan	H4	K12	Increased expression of ABC transporters promote the transition of colorectal cancer cells into a diapause‐like state	Resistance	[54]
	Pemetrexed	H4	K12	Activate transcription of *CCNB1*, accelerate DNA replication and the cell cycle	Resistance	[56]
	5‐Fu	CEACAM6	K12	Promote the proliferation of colorectal cancer cells	Sensitivity	[53]
	Temozolomide	H3	K9	Activate the transcription and expression of *LUC7L2*, reduce MLH1 expression, and inhibit mismatch repair	Resistance	[44]
Targeting drug	Bevacizumab	H3	K18	Promote autophagy and cell survival in hypoxic stress	Resistance	[73]
	Proteasome inhibitor (PI)	IGF‐1R		Activate MET and inhibit cuproptosis	Resistance	[66]
	All‐*trans* retinoic acid (ATRA)	Histone and METTL3		Overexpression of METTL3	Resistance	[57]
Immunotherapy	CAR‐T cell treatment	H3	K18	Enhance the expression of CD39, CD73, and CCR8	Resistance	[93]
	T cell‐related immunotherapy	Histone		Promote the immunosuppressive activity of MDMs and increase IL‐10 expression	Resistance	[114]
	4‐1BB agonist antibody			Induce CD8^+^T cells	Sensitivity	[114]
	PD‐1/PD‐L‐1 inhibitor	Histone	H3K18, H4K5, H4K8, and H4K12	Histone lactylation induces PD‐L1 transcription	Sensitivity	[95]
	ADT/PI3Ki combination therapy	H3	K18	Promote the transition to an immunosuppressive phenotype in tumors	Sensitivity	[109]
	ADT/PI4Ki combination therapy	H3	K18	Crosstalk between tumor cells and TAMs	Sensitivity	[112]
	—	H3, METTL3	H3K18, METTL3 zinc finger domain	Promote the immunosuppressive ability of tumor‐infiltrating myeloid cells	—	[106]

### 3.1 Lactylation and Chemotherapy

The impact of lactate metabolism on the efficacy of chemotherapeutic drugs can be reflected at three levels, from small to large: lactylation modification of intracellular proteins, intracellular lactate levels, and lactate levels in the TME. This poses a holistic challenge to cancer chemoresistance, while simultaneously providing multiple avenues for therapeutic solutions.

#### 3.1.1 Lactate Modification of Intracellular Proteins

**FIGURE 2:**
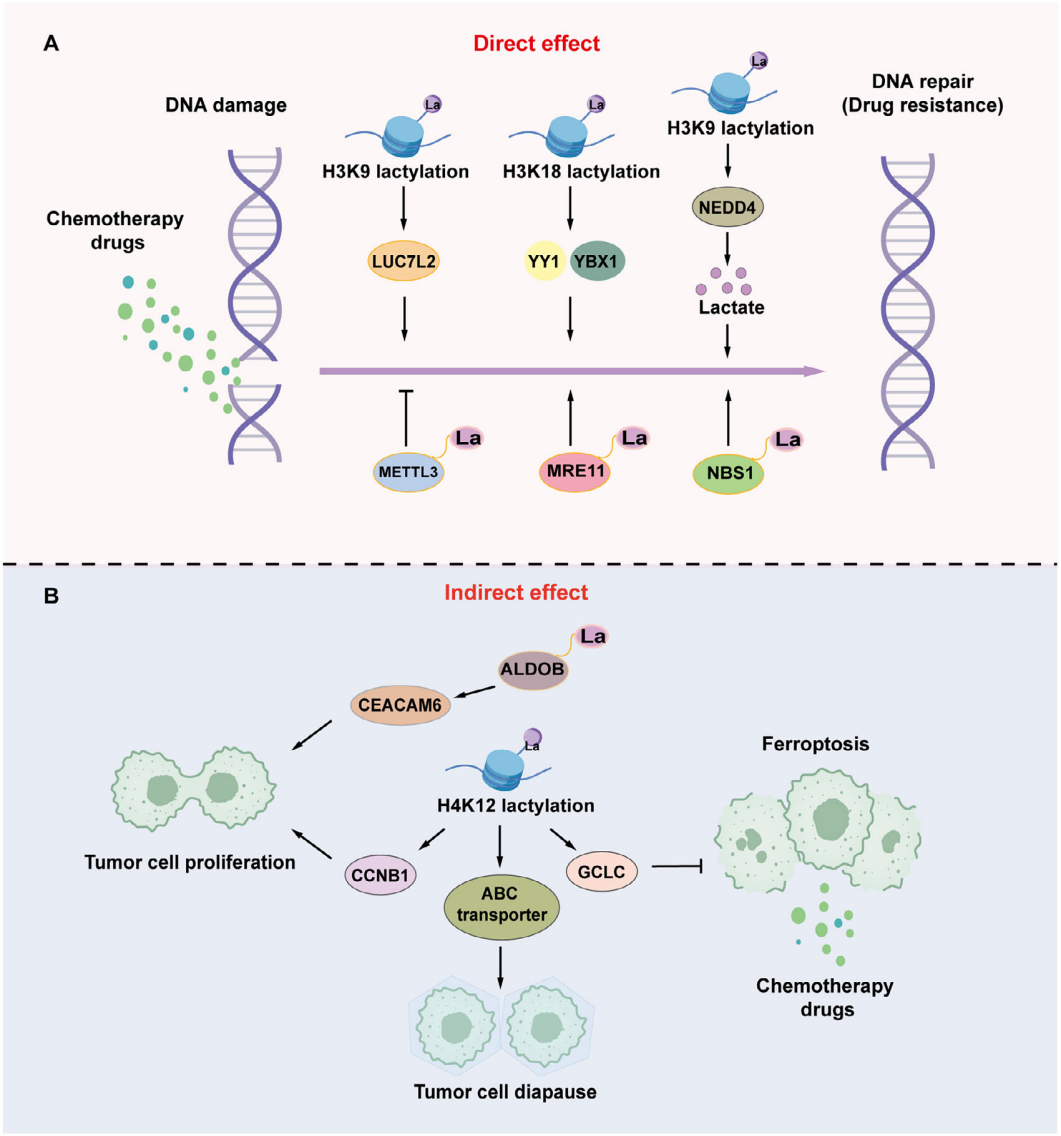
Lactate and lactylation regulate chemotherapy sensitivity through direct and indirect mechanisms. (A) Lactate and lactylation directly affects chemotherapy sensitivity by modifying and regulating key functional proteins in the chemotherapy drug action pathway. (B) An indirect effect is exerted by promoting cell proliferation, inducing cell quiescence, or inhibiting ferroptosis and other biological processes. Arrows represent promotion and termination lines represent inhibition.

Building on this metabolic‐epigenetic nexus, lactylated proteins profoundly influence chemotherapy sensitivity through two primary pathways: directly regulating DNA repair related to chemotherapy drugs or indirectly leading to drug resistance by promoting cell proliferation and inhibiting programmed cell death (Figure 2).

A deep layer of resistance emerges from hyperactive DNA repair pathways, where lactylation directly enhances DNA repair efficiency. Highly active homologous recombination repair often underlies chemoresistance, as demonstrated by Chen et al. who found that MRE11 lactylation at K673 strengthens its DNA binding activity, facilitating end resection and recombination, thereby conferring broad chemoresistance to breast and colon cancers [11, 40, 41]. In cisplatin‐resistant triple‐negative breast cancer cells, histone deacetylase 2 (HDAC2) mediates METTL3 delactylation, promoting N6‐methyladenosine (m6A) modifications of DNA damage repair‐related transcripts and enhancing cell survival under cisplatin treatment [42, 43]. H3K9la enrichment in glioblastoma activates *LUC7L2* transcription, leading to *MLH1* intron retention, reduced MLH1 expression, and impaired mismatch repair, ultimately driving temozolomide (TMZ) resistance [44, 45]. Additionally, NBS1 lactylation at K388 is critical for cisplatin resistance in NSCLC, as it stabilizes the MRE11‐RAD50‐NBS1 complex and accumulates repair proteins at DNA double‐strand breaks [12, 46, 47].

Lactylation modifications also serve as a crucial bridge between the tumor metabolic environment and epigenetic alterations, and play a pivotal role in indirectly fostering tumor chemoresistance by integrating glycolytic flux with gene regulation and cellular survival mechanisms. This interplay is exemplified in CRC stem cells, where p300‐mediated H4K12la and HDAC1‐mediated delactylation upregulates the expression of GCLC, a key enzyme in glutathione (GSH) synthesis that inhibits ferroptosis and thereby confers chemoresistance in CRC stem cells. These mechanisms underscore how lactylation sustains metabolic reprogramming and shields cancer cells from oxidative stress‐induced death, highlighting a vulnerability that can be exploited for novel ferroptosis‐inducing therapies [48, 49]. In cisplatin‐resistant bladder cancer cells, enhanced glycolysis elevates histone lactylation, particularly at histone H3 lysine 18 (H3K18la), which enriches the promoter regions of resistance‐associated genes such as the transcription factors *YY1* and *YBX1*, thereby boosting their transcription [50]. This heightened activity of YY1 and YBX1 upregulates DNA repair genes, multidrug resistance transporters, and epidermal growth factor receptor signaling, culminating in broad chemotherapeutic resistance across various tumors [51, 52]. Similarly, in CRC, aldolase B (ALDOB)‐mediated lactylation activates the downstream effector carcinoembryonic antigen cell adhesion molecule‐6 (CEACAM6) via enhanced Kla, stabilizing CEACAM6 and amplifying glycolysis‐driven lactate production and secretion, which in turn promotes cell proliferation and resistance to 5‐fluorouracil (5‐FU) [53]. Furthermore, H4K12la in CRC elevates ABC transporter expression, inducing a diapause‐like state that diminishes sensitivity to irinotecan [54, 55]. This pattern extends to lung cancer brain metastases, where AKR1B10‐driven H4K12la, fueled by lactate dehydrogenase A (LDHA) and elevated lactate levels, activates *CCNB1* transcription to accelerate DNA replication and cell cycle progression, thereby conferring acquired pemetrexed resistance [56]. Moreover, studies have identified several lactylated proteins that are significantly associated with chemotherapy resistance; however, the underlying mechanisms remain unclear. For example, histone lactylation and METTL3 expression were significantly upregulated in all‐*trans* retinoic acid (ATRA)‐resistant acute promyelocytic leukemia cells [57]. These examples reveal that lactylation acts as a dynamic sensor of metabolic stress, not only amplifying repair capacity but also enabling tumors to adaptively proliferate and survive, which challenges the efficacy of chemotherapies.

#### 3.1.2 Lactate Level Modulation

Activated glycolysis and increased lactate levels modulate signaling pathways to further entrench drug resistance, revealing an intricate crosstalk between metabolism and ubiquitination. In hepatocellular carcinoma (HCC), lactate, a glycolytic substrate, fuels histone lactylation, which upregulates NEDD4 expression. NEDD4 then interacts with PTEN to induce its ubiquitination and degradation, thereby accelerating glycolysis and conferring resistance to oxaliplatin and 5‐FU [58, 59]. In ovarian cancer cells, the chemotherapy‐induced downregulation of glucose transporters activates ACAT1, leading to ME2 acetylation at K156 and robust lactate production, which sustains DNA damage repair and suppresses acquired chemoresistance‐targeting ACAT1, which effectively inhibits this process [60]. These findings highlight that tumors exploit chemotherapy‐induced metabolic shifts to hyperproduce lactate, perpetuating lactylation‐dependent signaling that undermines therapeutic efficacy, and suggesting that early metabolic interventions could prevent the development of treatment resistance. Beyond intracellular mechanisms, the TME contributes to lactate‐induced chemoresistance, where extracellular lactate reduces chemosensitivity and impairs therapeutic efficacy [61]. Lactate directly bolsters cancer cell resistance, and fluctuations in lactate levels may reprogram protein lactylation; however, the precise underlying mechanisms warrant further investigation [62]. This TME‐metabolism interface offers a critical insight: chemoresistance is not merely cell‐autonomous but ecosystem‐driven, implying that therapies disrupting lactate shuttles could synergize with existing regimens.

In summary, lactylation has emerged as a master regulator of chemotherapy resistance, seamlessly linking metabolic reprogramming to the epigenetic control of DNA repair and signaling pathways across diverse cancers. Intracellular lactate and lactate in the TME also manipulate the response to chemotherapeutic drugs. These insights not only illuminate why tumors evade treatment, but also pave the way for innovative strategies, such as combining lactylation inhibitors (e.g., targeting LDHA or HDACs) with chemotherapeutics, to dismantle adaptive barriers and improve patient outcomes in precision oncology.

### 3.2 Lactylation and Targeted Drugs

**FIGURE 3:**
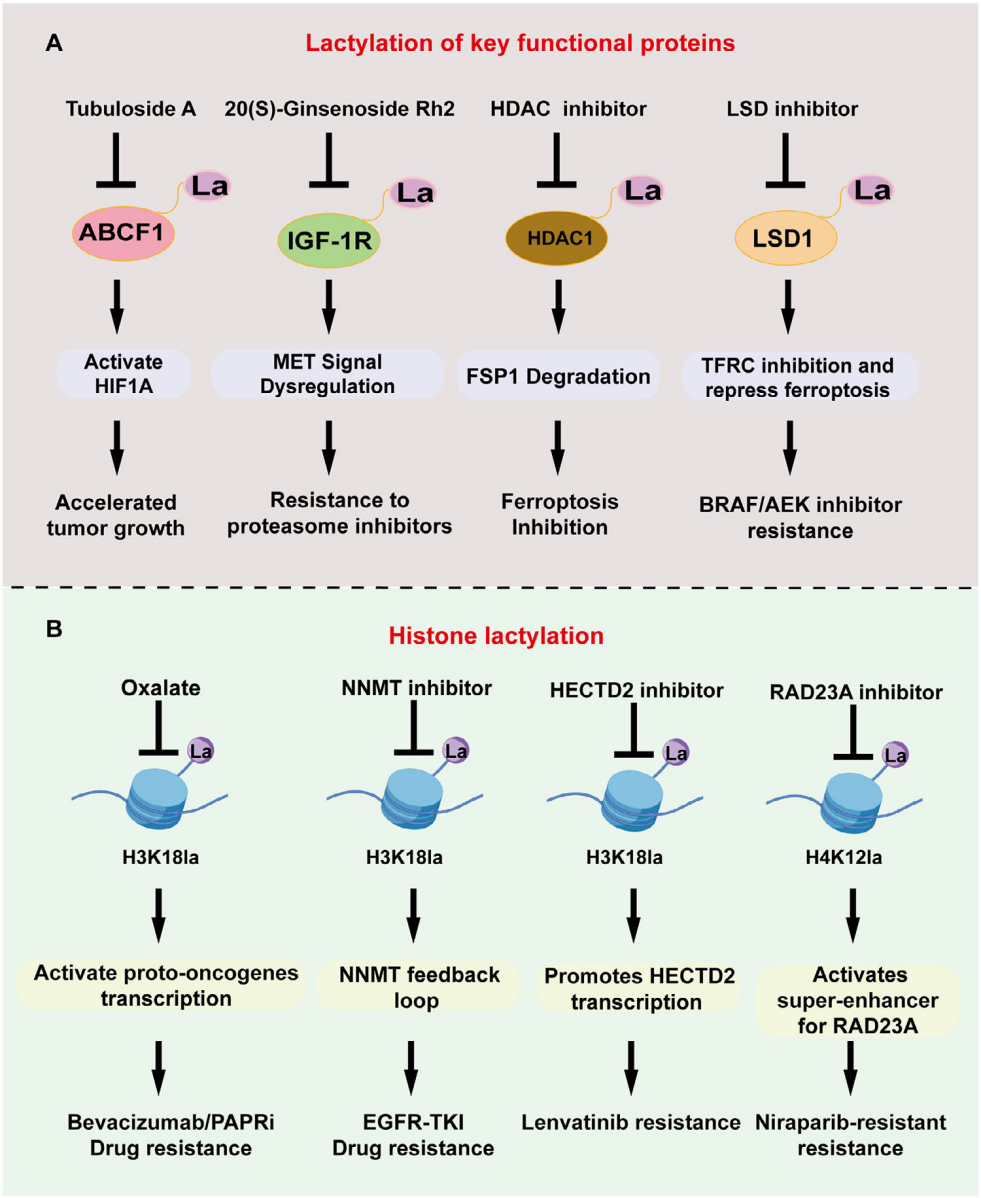
Lactylation modification regulates the response to targeted drugs in two aspects. (A) The first aspect is the lactylation modification of key functional proteins in the action of targeted drugs, which further regulates downstream pathways and leads to drug resistance or tumor progression. (B) The second aspect is the regulation of histone lactylation modification, which in turn alters the transcription of histone target genes and leads to drug resistance. Arrows represent promotion and termination lines represent inhibition.

Targeted drugs represent a paradigm shift in oncology. These drugs were designed to selectively inhibit specific oncogenic genes or proteins, achieving high tumor concentrations while minimizing toxicity to normal cells, thereby enabling precise cancer treatment with superior efficacy and reduced side effects compared to traditional chemotherapy [63, 64]. This precision is particularly evident in their ability to spare healthy tissues; however, resistance mechanisms pose significant challenges. Lactylation influences the efficacy of targeted therapies via two principal mechanisms (Figure 3). First, it mediates the PTM of key nonhistone proteins involved in the signaling pathways targeted by therapeutic agents. This modification can alter protein function, stability, or their interactions with other molecules, thereby modulating downstream signaling cascades and potentially diminishing drug effectiveness, ultimately contributing to the development of drug resistance. Second, histone lactylation regulates chromatin structure and gene transcription, often resulting in broad transcriptional changes. Collectively, these mechanisms underscore the role of lactylation as a significant regulatory factor in the response to targeted treatments.

Lactylation targets key nonhistone proteins that are directly or indirectly involved in the mechanism of action of targeted therapeutics. By adding lactyl groups to specific lysine residues on these proteins, lactylation can alter their stability, subcellular localization, enzymatic activity, or protein–protein interactions. Such functional changes may interfere with the intended drug‐target interaction, impair downstream signaling inhibition, or activate compensatory pathways, ultimately contributing to the development of drug resistance. In HCC, ABCF1‐K430 lactylation (ABCF1‐K430la) activates the histone demethylase KDM3A by removing the repressive H3K9me2 mark, leading to the transcriptional activation of *HIF1A* and accelerated tumor growth under hypoxia. Tubulin A, a natural inhibitor of this lactylation, has emerged as a promising therapeutic agent, positioning ABCF1‐K430la as both a prognostic biomarker and target [65]. Similarly, in multiple myeloma, MUC20 suppresses IGF‐1R lactylation in proteasome inhibitor‐resistant cells, inhibiting MET activation and inducing cuproptosis via CDKN2A downregulation, thereby attenuating resistance [66, 67, 68, 69]. Meanwhile, 20(S)‐ginsenoside Rh2 (GRh2), a natural deacetylase inhibitor, reverses this by lowering lactylation and inhibiting METTL3, offering a novel strategy to overcome resistance [57]. Histone deacetylase inhibitors (HDACi) like vorinostat (SAHA) and Trichostatin A (TSA) enhance CRC ferroptosis susceptibility by reducing HDAC1K412la, promoting *FSP1* mRNA degradation via H3K27ac on m6A erasers, suggesting synergistic HDACi‐ferroptosis inducer strategies [70]. In BRAFi/MEKi‐resistant melanoma, lactate induces LSD1 lactylation, stabilizing FosL1 to repress ferroptosis via TFRC inhibition; combined therapy with LSD1 inhibitors and immunotherapy overcomes resistance [71, 72]. These examples illustrate that lactylation not only amplifies hypoxic adaptation, but also creates actionable vulnerabilities for small‐molecule interventions in metabolically stressed tumors.

The second mechanism is mediated by histone lactylation, which modulates gene expression by altering chromatin structure and transcriptional activity. This epigenetic marker is typically associated with active transcription, particularly at the promoters and enhancers of genes involved in cell adaptation, immune modulation, and stress responses. Histone lactylation can induce a broad and sustained drug resistance phenotype in cells by promoting the expression of pro‐survival genes, multidrug resistance transporters, and antiapoptotic factors. Moreover, as this form of regulation simultaneously affects multiple genes, its impact is more systemic and long‐lasting than that of modifications targeting individual proteins. Extending this metabolic‐epigenetic interplay to anti‐angiogenic therapies such as bevacizumab, a cornerstone of the first‐line treatment of metastatic CRC, faces resistance linked to elevated histone lactylation levels. For example, H3K18la promotes RUBCNL/Pacer transcription, facilitating autophagosome maturation through interactions with beclin 1 (BECN1) and the recruitment of PIK3C3/VPS34, which sustains cell proliferation and survival in hypoxic cancer cells. Preclinical models using cells from treatment‐resistant patients have demonstrated that combining oxalate, a lactylation inhibitor, with bevacizumab yields superior efficacy, highlighting that targeting lactylation can restore sensitivity by disrupting autophagy‐dependent survival [73, 74]. In EGFR‐mutated NSCLC, acquired EGFR‐tyrosine kinase inhibitor (TKI) resistance (e.g., gefitinib, erlotinib, and osimertinib) involves elevated levels of NNMT, which depletes methyl donors, reducing H3K9me3/H3K27me3 and activating EGR1 and ALDH3A1. In turn, ALDH3A1 boosts lactate, stimulating NNMT via p300‐mediated H3K18la, forming feedback loops; this mechanism shows the synergy between NNMT inhibitors and TKIs [75]. In HCC, H3K18la promotes the transcription of *HECTD2*, an E3 ligase that degrades KEAP1 to activate NRF2 antioxidative responses, thereby attenuating the response to lenvatinib; targeting HECTD2 via nanoparticles to overcomes treatment resistance [76]. In niraparib‐resistant ovarian cancer, glycolytic activation results in the accumulation of lactate, promoting H4K12la that, via MYC, activates a super‐enhancer for RAD23A expression, correlating with pyruvate kinase M2 (PKM2) and LDHA‐enhancing DNA damage repair and resistance; thus, RAD23A is a potential therapeutic target [77]. Collectively, these cases reveal key insights: histone lactylation acts as a dynamic modulator of oncogenic signaling, and its inhibition can repurpose existing targeted therapies by exploiting metabolic dependencies.

While previous studies have predominantly focused on upstream pathways in lactylation‐mediated targeted drug resistance, emerging evidence suggests a direct impact on drug targets themselves, potentially altering protein conformations and binding affinities. For example, the lactylation of α‐myosin heavy chain (α‐MHC) at K1897 enhances interactions with titin, improving cardiac function and ameliorating heart failure [78, 79]. We speculate that similar modifications in drug targets could impair inhibitor binding, fostering resistance. Moreover, lactate itself is intricately linked to targeted drug responses, inducing resistance to AKT inhibitors in colon cancer cells and prompting abundant lactate release into the TME in TKI‐resistant cancers [62, 80]. Thus, lactate‐mediated metabolic symbiosis resists the efficacy of antiangiogenesis agents and TKIs through intercellular lactate shuttles [81]. Future research into the lactate or lactylation regulatory mechanisms of these targets may unveil novel insights, with drugs addressing both their targets and their lactylation states, paving new paths for precision medicine. This shift in focus underscores the deeper implication that the conformational effects of lactylation could explain the elusive resistance patterns observed in certain malignancies.

In summary, lactylation profoundly shapes resistance to targeted drugs by orchestrating metabolic‐epigenetic adaptations across cancers, from hypoxia signaling in HCC to DNA repair in ovarian cancer and ferroptosis suppression in CRC and melanoma. These insights not only reveal lactylation as a convergence point for diverse resistance mechanisms, but also advocate for integrated approaches, such as combining lactylation inhibitors with existing targeted agents to disrupt feedback loops and enhance precision medicine, ultimately transforming outcomes in treatment‐resistant tumors.

### 3.3 Lactylation and Immunotherapy

**FIGURE 4:**
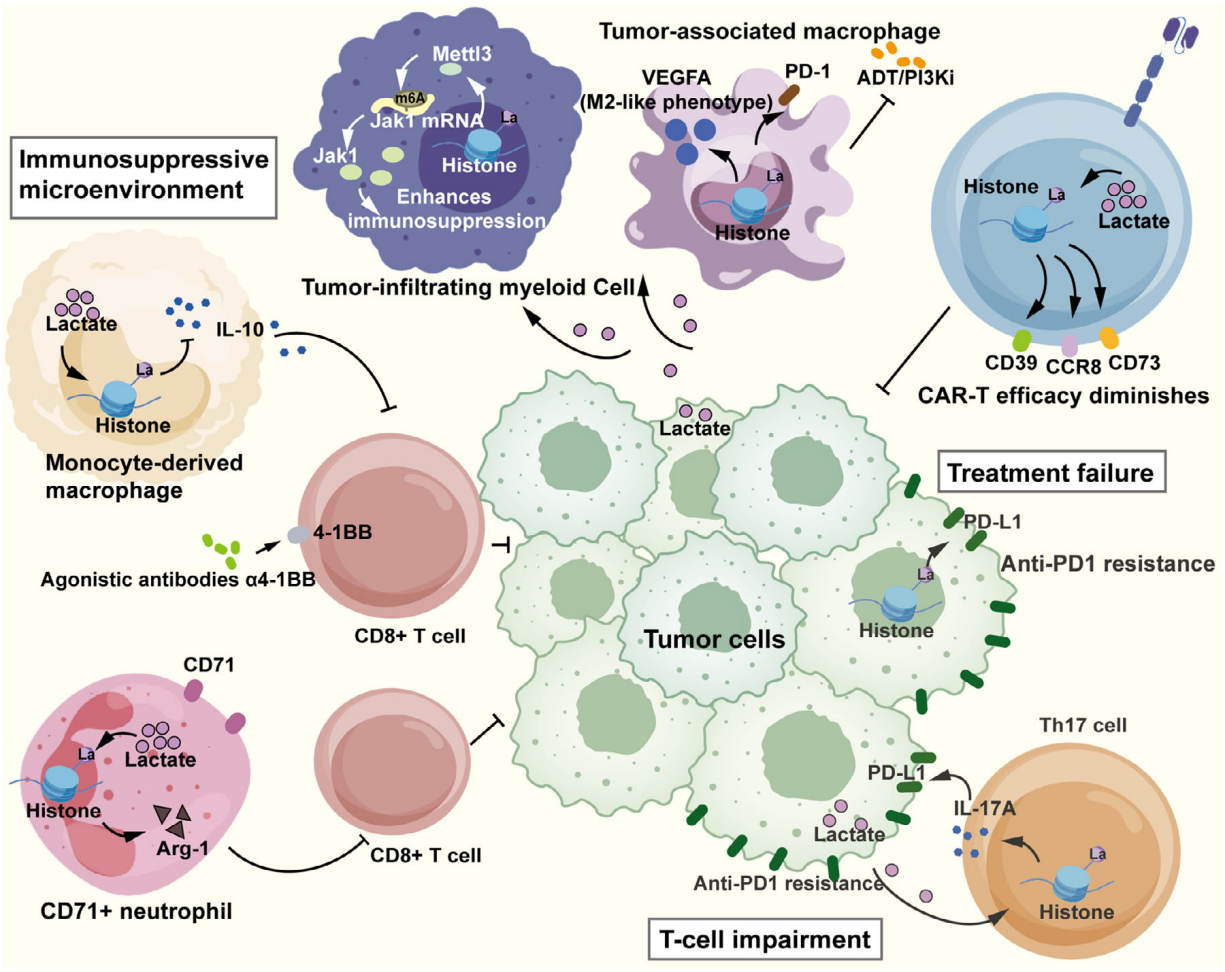
Specific mechanisms how lactate and lactylation regulate the response to immunotherapy. Lactate and lactylation modifications in CAR‐T cells or tumor cells can directly contribute to immunotherapy failure. Additionally, the promotion of an immunosuppressive tumor microenvironment and the lactylation‐mediated impairment of T cell function drive tumor progression and confer resistance to immunotherapy. Arrows represent promotion and termination lines represent inhibition.

Metabolic reprogramming in cancer cells can subvert immune surveillance mechanisms. The accumulation of lactate and subsequent protein lactylation profoundly alters the functional properties of both tumor and immune cells, thereby promoting immune evasion and contributing to the formation of an immunosuppressive TME (Figure 4). Furthermore, metabolite sensors and lysine lactyltransferases have emerged as key regulators that mediate the effects of lactate on immune function, serving as molecular links between metabolic signals and epigenetic modifications that influence disease progression.

Tumor immunotherapy has evolved from the “releasing the brake” to the “stepping on the gas” phase. Now, the goal has been to amplify the tumor‐killing activity of immune cells, shifting away from reducing immunosuppression. The primary approaches for this goal include employing pharmaceuticals to boost the ability of immune cells to destroy tumors within the patient's body, and the genetic engineering of T cells ex vivo to enhance their efficacy. Among the most prevalent cellular therapies, CAR‐T cell therapy selectively targets and eliminates tumor cells by modifying the patient's own T cells. However, despite their potential, CAR‐T cells can induce toxic reactions and develop resistance during after treatment [82, 83, 84, 85]. In glioblastoma, histone H3K18 lactylation directly increases the activity of gene promoters, such as CD39, CD73, and CCR8, and promotes their transcriptional expression, diminishing the effectiveness of CAR‐T therapy [86, 87, 88, 89]. The combination of oxalate and CAR‐T therapy not only reprograms the glucose metabolism of cancer cells but also alters the immunosuppressive TME by reducing adenosine production and the tumor infiltration of regulatory T (Treg) cells [90, 91, 92]. The inhibition of lactate production by oxalate and lactylation enhances CAR‐T cell function in glioblastoma therapy [93]. On the other hand, regarding the “releasing the brake” paradigm, drug immune checkpoint inhibitors have been successfully used in a wide range of cancers, including hematologic malignancies. However, lactate accumulation and modification may interfere with drug efficacy in this context. For example, STAT5, which is highly expressed in acute myeloid leukemia (AML), promotes glycolysis in AML cells and the nuclear translocation of E3BP, facilitates histone lactylation, and ultimately induces *PD‐L1* transcription [94], ultimately contributing to immunosuppression in AML. These effects are significant in the context of PD‐1/PD‐L1 interactions with reactive CD8^+^ T cells, which are influenced by immune checkpoint inhibitors [95, 96, 97].

The regulation of lactylation may influence the therapeutic efficacy of drugs by regulating the function of immune cells within the immune microenvironment [98, 99]. In cocultures of arsenite‐treated HepG2 (HCC cell line) and Jurkat T (human T lymphocyte leukemia) cells, hepatocyte‐derived lactate induces histone H3 lysine 18 lactylation (H3K18la), an epigenetic modification that upregulates interferon regulatory factor 4 (IRF4) and retinoic acid receptor‐related orphan receptor gamma t (RORγt) expression, thereby promoting Th17 cell differentiation [100, 101]. Differentiated Th17 cells secrete high levels of interleukin‐17A (IL‐17A), which contributes to resistance to anti‐PD‐1 therapy by enhancing PD‐L1 expression in patients with various cancers [102, 103, 104, 105]. These findings provide insights into the role of lactylation in clinical decision‐making regarding T cell fate and anticancer immunotherapy. Beyond T cells, the lactate‐mediated regulation of other immune cells contributes to the formation of an overall immunosuppressive TME. It has been confirmed that lactate accumulation in the TME induces the upregulation of METTL3 in TIMs via the lactylation of H3K18; two direct lactylation modification sites have been identified in the zinc‐finger domain of METTL3. The expression of METTL3 in TIMs is associated with poor prognosis in colon cancer. METTL3 promotes the m6A modification of *Jak1* mRNA, the m6A‐YTHDF1 axis enhances Jak1 translation efficiency, and subsequent STAT3 phosphorylation enhances the immunosuppression of TIMs [106, 107]. Lactylation has also been reported to impact macrophage polarization [108]. In aggressive variant prostate cancer, lactate accumulation in the TME promotes histone lactylation within tumor‐associated macrophages (TAMs), shifting them to an immunosuppressive phenotype. Combination treatment with androgen deprivation therapy (ADT)/PI3K inhibitors (PI3Ki) was inhibited by the recruitment of PD‐1‐expressing TAMs [109, 110, 111]. To improve the anticancer efficacy of the combination of the PI3K/AKT pathway and ADT, Kiranj et al. achieved durable ADT‐independent tumor control by disrupting the lactate‐mediated crosstalk between cancer cells and TAMs and inhibiting histone lactylation (H3K18lac) within TAMs [112]. Lactate induces histone lysine lactylation to promote macrophage polarization toward an M2‐like phenotype, which is characterized by the secretion of VEGFA, and consequently suppress the immune response within the TMEs of melanoma and lung cancer [7]. Furthermore, 4‐1BB is a T cell co‐stimulatory molecule primarily expressed on activated CD4^+^ and CD8^+^ T cells and natural killer cells. The agonistic antibodies α4‐1BB can induce CD8^+^ T cells to release more effector molecules, increase their proliferation, and reduce their apoptosis [113]. Lactate‐driven regulation may significantly influence the activation and cytotoxic capabilities of immune cells, thereby representing a potential therapeutic target. High glycolysis and lactate production in monocyte‐derived macrophages (MDMs) are promoted by microglia in glioblastoma multiforme. The PERK‐ATF4‐GLUT1 axis promotes histone lactylation, and intracellular lactate‐driven histone lactylation inhibits IL‐10 expression and T cell activity. The absence of PERK in MDMs leads to the cessation of histone lactylation, promoting intratumoral T cell accumulation, delaying tumor growth, and potentially enhancing the effects of α4‐1BB in blocking glioblastoma progression [114]. Under hypoxic conditions, CD71^+^ neutrophils engage in glycolysis to produce substantial amounts of lactate. Lactate then induces histone lactylation, promoting the expression of arginase‐1 (Arg‐1), and leading to the suppression of T cell function [18, 115]. Isosafrole, an antiepileptic drug, has been shown to effectively inhibit histone lactylation. In murine models of brain tumors, isosafrole delays tumor growth and resensitizes immunotherapy‐resistant tumors to immune checkpoint blockade [116].

Trained immunity represents the innate immune system's capacity for long‐term functional reprogramming (“memory”). Trained monocytes preferentially utilize lactate as a tricarboxylic acid (TCA) cycle substrate. Lactate further modulates the trained immunity by driving histone lactylation, an epigenetic reprogramming mechanism. As a central immunometabolite of trained immunity, lactate deepens our understanding of innate immune memory and unveils novel therapeutic avenues [117].

Moreover, lactate‐related genes and lactylation regulators may influence immune escape and play important roles in the immunosuppressive TME. For example, metabolite sensors play a pivotal role in regulating immune responses. Lactate functions as a signaling molecule and influences immune cell function through interactions with specific sensors. Key enzymes, including AARS1 and AARS2, serve as intracellular lactate sensors and lysine lactyltransferases that catalyze the lactylation of various proteins, such as cGAS. By modulating cGAS activity, lactate governs the balance between immune tolerance and inflammation, particularly in diseases such as cancer and chronic inflammatory disorders [118, 119, 120, 121].

Utilizing the lactate‐associated genes identified through an integration of 108 algorithmic combinations, patients were stratified into low‐ and high‐risk groups, exhibiting significant differences in survival outcomes. The low‐risk group showed an elevated expression of immune checkpoint molecules and enhanced immune cell infiltration, suggesting a potentially enhanced response to immunotherapy. Conversely, the high‐risk group displayed immunosuppressive features associated with a poorer prognosis. This model holds promise as a potential prognostic biomarker for personalized breast cancer therapy [122].

In summary, lactate and lactylation play significant roles in tumor immune escape and the establishment of an immunosuppressive environment. Numerous studies have confirmed that altering the tumor immune cell phenotype and immunosuppressive status through lactylation can enhance the efficacy of cellular therapies. This insight offers a new therapeutic strategy and thought process for patients who are unresponsive to conventional therapies.

## 4 Factors Affecting Therapeutic Efficacy by Modulating Lactylation

We hypothesize that the regulation of lactylation could be an ideal solution for cancer treatment. Here, we summarize multiple factors affecting lactylation, including other PTMs and metabolic reprogramming mechanisms, to provide new ideas for targeted lactylation therapies in the future.

### 4.1 PTMs Affect Lactylation Modifications

PTMs represent the cornerstone of proteomic diversity and functional regulation, profoundly influencing cellular responses to environmental cues [123, 124, 125, 126]. PTMs expand the functional repertoire of the proteome via the covalent addition of functional groups, proteins, or proteolytic events, such as phosphorylation, glycosylation, ubiquitination, nitrosylation, methylation, acetylation, lipidation, and cleavage [127]. These modifications transcend genomic constraints and modulate protein activity, subcellular localization, and interactions with nucleic acids, lipids, and cofactors [128]. Among these, lactylation/lactate‐derived lysine modification has emerged as a dynamic epigenetic marker that intersects with canonical PTMs such as phosphorylation, acetylation, and ubiquitination through mechanisms of site competition, enzymatic crosstalk, and metabolic reprogramming. This interplay not only amplifies the proteome's adaptive capacity, but also drives pathological states, including cancer progression, by linking glycolytic fluxes to gene expression and protein stability.

In the context of lactylation, the influence manifests primarily through two axes: direct competition at shared lysine residues, and indirect modulation of lactylation‐mediated functions. For instance, acetylation and ubiquitination, which target the ε‐amino groups of lysine, can sterically or enzymatically compete with lactylation, steering proteins toward distinct fates such as transcriptional activation versus proteasomal degradation. Non‐lysine‐associated but not site‐overlapping PTMs, such as phosphorylation, indirectly influence lactylation by altering glycolytic enzyme kinetics and lactate availability.

Phosphorylation, which entails the transfer of γ‐phosphate from ATP/GTP to serine, threonine, or tyrosine residues by kinases, is a quintessential reversible switch in signal transduction events [129, 130]. Its ubiquity in modulating cell proliferation, differentiation, and apoptosis [129, 131, 132] extends to lactylation via the upstream regulation of lactate biosynthesis. Phosphorylation of the glycolytic gatekeeper LDHA is central to this process. For example, FKBP10, a chaperone implicated in ECM remodeling, binds directly to the C‐terminal domain of LDHA, facilitating its phosphorylation at tyrosine‐10 (Y10). This posttranslational event hyperactivates the Warburg effect in clear cell renal cell carcinoma (ccRCC), elevating lactate and histone lactylation to fuel hypoxic adaptation and therapeutic resistance to HIF2α inhibitors [133]. Under the activation of ULK1, LDHA is phosphorylated during nutrient deficiency, promoting lactate production. Moreover, lactate links autophagy and glycolysis through the lysine lactylation of Vps34 (at positions K‐356 and K‐781), enhancing the binding of Vps34 to Beclin1, Atg14L, and UVRAG, increasing lipid kinase activity, and thereby promoting autophagic flux and endolysosomal transport [134]. This mechanism maintains cellular homeostasis in the skeletal muscles during intense exercise and is associated with cancer progression [135].

Acetylation, which is mediated by lysine acetyltransferases (KATs) using acetyl‐CoA as a donor and reversed by lysine deacetylases (KDACs), is indispensable for transcriptional fidelity, cell cycle progression, DNA repair, and cytoskeletal dynamics [125, 136, 137, 138, 139]. Dysregulated acetylation fosters oncogenic hallmarks, including metabolic rewiring and microenvironmental adaptation [140]. Lactylation and acetylation converge strikingly, sharing lysine substrates, writers (e.g., p300/CBP), and erasers (e.g., HDACs and SIRTs), fostering competitive antagonism rather than hierarchical regulation. This suggests that acetylation and lactylation have a more intimate connection, unlike phosphorylation‐related regulatory mechanisms where acetylation and lactylation are likely to be horizontally linked. In hepatic stellate cells (HSCs), hexokinase 2 (HK2) induction is required for lactylation‐mediated gene expression during fibrosis; however, it competes with acetylation. Class I HDAC inhibitors abrogate HSC activation by favoring acetylation over lactylation, underscoring eraser‐mediated rivalry [141]. Moreover, downregulation of the deacetylase sirtuin 3 (SIRT3) mediates the hyperacetylation and inactivation of pyruvate dehydrogenase E1 component subunit alpha (PDHA1) in sepsis‐induced acute kidney injury, leading to lactate overproduction in renal tubular epithelial cells. Lactate also mediates the lactylation of Fis1 at lysine 20 (Fis1 K20la). Elevated levels of Fis1 K20la promote mitochondrial proliferation, which in turn causes ATP depletion, excess production of mitochondrial reactive oxygen species, and mitochondrial apoptosis [142]. Thus, the acetylation‐lactylation crosstalk oscillates between rivalry and synergy, with metabolic perturbations dictating dominance in disease contexts.

A seminal advancement in dissecting the lactylation landscape involves distinguishing its isomers: lysine L‐lactylation (Kla), the predominant histone‐associated form; N‐ε‐(carboxyethyl)‐lysine (Kce); and D‐lactyl‐lysine (Kd‐la). Analytical pipelines leveraging mass spectrometry and enzymatic assays have established Kla as a glycolysis‐driven isomer, with lactyl‐CoA levels positively correlated with its abundance, thereby providing a robust framework for isomer‐specific quantification [8, 143]. This methodological precision highlights the metabolic fidelity of lactylation, yet it also raises intriguing hypotheses: other PTMs may indirectly govern lactylation by reshaping protein conformations or trafficking, potentially occluding writer/eraser access, or altering lactate microenvironments. These avenues pave the way for more structural biology investigations.

### 4.2 Factors Affecting Lactylation Modifications via Metabolic Reprogramming

Tumor metabolic reprogramming, particularly the dysregulated surge in glycolysis, profoundly modulates lactylation by perturbing lactate homeostasis or fine‐tuning pivotal enzymatic cascades, thereby promoting tumor progression and adaptive resilience. Conversely, lactylation imprints metabolic rewiring by posttranslationally influencing key metabolic effectors, such as fatty acid synthase, which drives aberrant hepatic lipid deposition, exacerbating steatosis and oncogenic lipid signaling [144, 145, 146]. This bidirectional crosstalk underscores a dynamic metabolic‐epigenetic axis, where excess glycolytic fuel (lactyl‐CoA) etches pro‐tumorigenic histone marks, while lactylation sustains the biosynthetic demands for proliferation and invasion.

**FIGURE 5:**
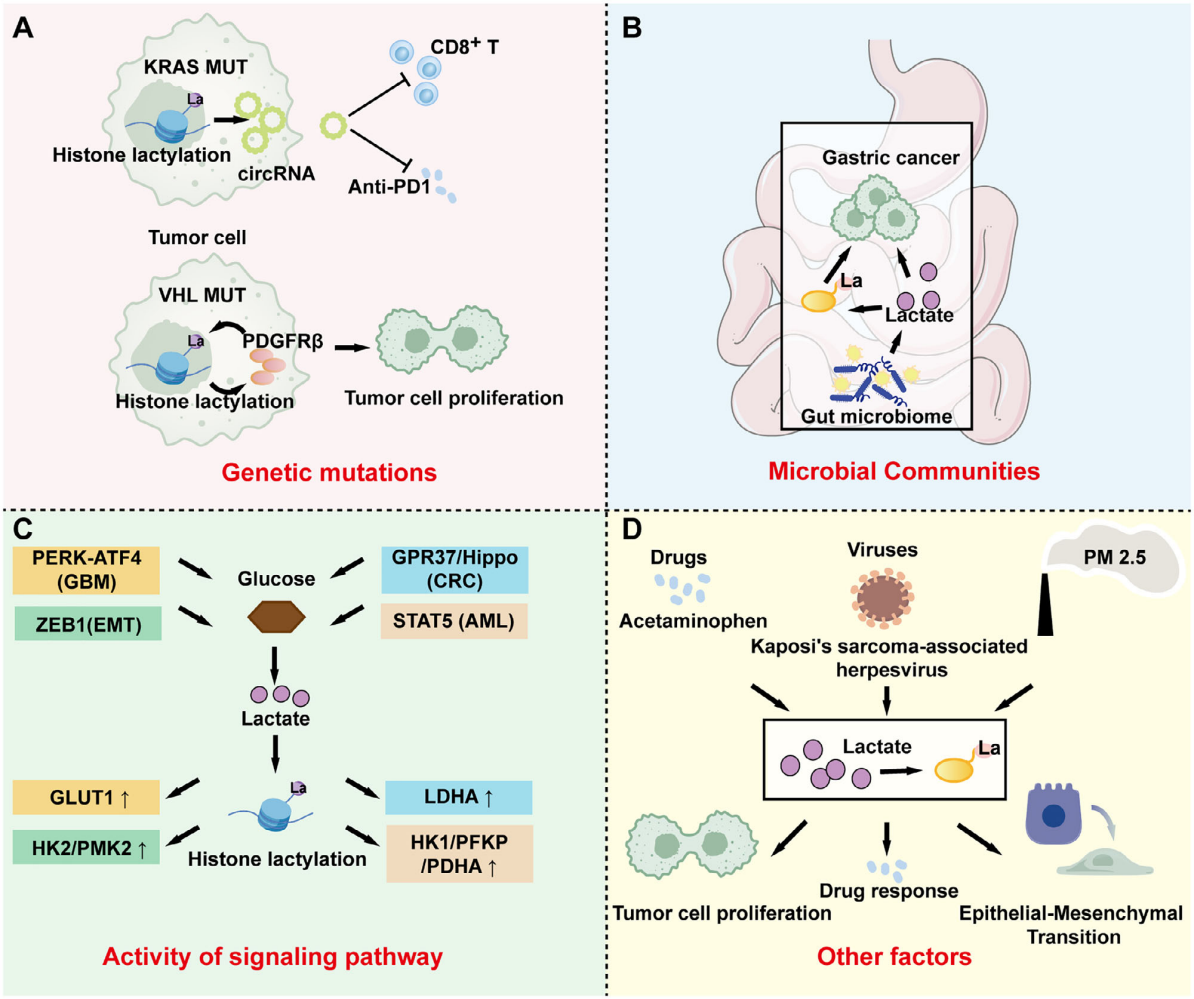
Factors affecting lactylation modifications via metabolic reprogramming. (A) Genetic mutations, such as KRAS mutations in tumor cells, lead to circRNA‐mediated CD8^+^ T cell inhibition and anti‐PD1 treatment resistance. Moreover, VHL mutations promote tumor cell proliferation via PDGFRβ signaling. (B) Microbial communities also affect lactylation. Shown here is the role of the gut microbiome in gastric cancer progression through lactate (La) production and secretion, facilitating microbial interactions with tumor cells. (C) Activity of signaling pathways. Highlighted here is how pathways converge on glucose metabolism to enhance lactate levels and histone lactylation, subsequently affecting the expression of downstream genes. (D) Other factors, including drugs, viruses, and environmental pollutants (e.g., PM2.5), collectively drive outcomes like tumor cell proliferation, altered drug responses, and epithelial‐mesenchymal transition (EMT) through lactate‐mediated mechanisms. Arrows represent promotion and termination lines represent inhibition.

Such reprogramming is induced by the interplay between intrinsic and extrinsic cues. Intrinsic drivers include oncogenic mutations and hyperactive intracellular signaling pathways that hijack glycolytic flux to evade apoptosis and fuel biomass accrual. Extrinsic modulators, including dysbiotic microbiota, pharmacological interventions, viruses, and environmental stressors (e.g., hypoxia or pollutants), impose systemic pressures that amplify lactate gradients and lactylation propensity, fostering tumor heterogeneity and therapeutic recalcitrance (Figure 5). This reciprocal loop positions lactylation as a metabolic sentinel, bridging the tumor‐intrinsic environment with niche‐imposed exigencies.

#### 4.2.1 Genetic Mutations

Mutations in oncogenes and tumor suppressor genes are frequently linked to adverse prognoses and diminished drug responses in various cancers, often through intricate mechanisms that reprogram cellular metabolism and the epigenetic landscape [147]. Emerging evidence indicates that specific mutations can orchestrate metabolic shifts toward enhanced glycolysis and lactate production, thereby driving lactylation modifications. This metabolic‐epigenetic crosstalk not only sustains tumor growth and immune evasion, but also confers resistance to therapies, highlighting lactylation as a reversible therapeutic target in contrast to irreversible genetic aberrations [148]. A paradigmatic example is KRAS‐mutant (KRAS^MUT^) tumors, which are prevalent in cancers such as colorectal, pancreatic, and lung adenocarcinomas, where these mutations exacerbate poor prognosis and immunotherapy resistance. In KRAS^MUT^ tumor cells, heightened glycolytic flux generates excess lactate, which induces histone lactylation at specific loci. This modification directly activates the transcription of circATXN7, which subsequently interacts with the NF‐κB p65 subunit, sequestering it in the cytoplasm and preventing its nuclear translocation. Consequently, this dampens T cell activation and sensitizes tumor‐specific cytotoxic T lymphocytes to activation‐induced cell death, fostering an immunosuppressive microenvironment that reduces immune infiltration and impairs the efficacy of anti‐PD‐1/PD‐L1 immunotherapies [149, 150, 151]. Targeting circATXN7 or its upstream lactylation in CD8^+^ T cells can selectively inhibit KRAS^MUT^‐driven tumors while synergizing with checkpoint blockade, potentially transforming the therapeutic landscape for historically recalcitrant malignancies. Another compelling case involves mutations in the Von Hippel–Lindau (VHL) tumor suppressor gene, which underlies the rare autosomal dominant hereditary disorder VHL disease and plays a pivotal role in ccRCC pathogenesis. Inactive VHL is vital for ccRCC pathogenesis and is associated with metabolic reprogramming [152]. Inactive VHL, resulting from loss‐of‐function mutations, disrupts the ubiquitin‐mediated degradation of hypoxia‐inducible factors (HIFs), leading to pseudohypoxic metabolic reprogramming characterized by aerobic glycolysis and lactate accumulation [153, 154]. Yang et al. demonstrated a positive correlation between inactive VHL and histone lactylation in patients with ccRCC, where high levels of histone lactylation were indicative of poor prognosis [155]. Inactive VHL‐triggered histone lactylation and platelet‐derived growth factor receptor β (PDGFRβ) create a positive feedback loop that promotes cancer progression: histone lactylation activates PDGFRβ transcription, which in turn stimulates further histone lactylation. In addition, the combined inhibition of histone lactylation and PDGFRβ significantly enhances the therapeutic effect. Notably, the dual inhibition of lactylation (e.g., via LDHA blockers) and PDGFRβ significantly potentiates therapeutic responses in preclinical models, suggesting that disrupting this loop could overcome resistance to TKIs and immunotherapies in ccRCC. P53 is a target of the lactylation “writer” AARS1. Liquid–liquid phase separation, DNA binding, and transcriptional activation have been shown to be inhibited in tumor cells with p53 variants carrying persistently lactylated lysine residues. Furthermore, in patients with cancer carrying wild‐type p53, AARS1 expression and p53 lactylation are associated with poor prognosis. β‐alanine disrupts the binding of lactate to AARS1, which can reduce p53 lactylation and thus alleviate tumorigenesis in animal models [34, 156, 157].

In addition to canonical oncogene and tumor suppressor mutations, variations in lactylation regulators and specific lactylation sites can substantially influence tumor progression and treatment responsiveness. For instance, somatic mutations in key lactylation “writers,” such as p300/CBP, cause cells to be more sensitive to oxidative DNA damage and exhibit defective base excision repair [158].

Collectively, these findings highlight lactylation as a dynamic mutation‐driven nexus between metabolism and epigenetics, with profound implications in oncology. Unlike static genetic mutations, the reversibility of lactylation positions it as a druggable interface, where modulating its levels could disrupt adaptive resistance mechanisms and sensitize tumors to conventional therapies. This paradigm shift has encouraged the development of lactylation‐targeted agents, such as small‐molecule inhibitors of LDHA or synthetic lactyl‐CoA analogs, to personalize treatment regimens and improve survival in mutation‐driven cancers. Future research should prioritize the mapping of lactylomes in diverse mutational contexts to fully harness their therapeutic potential.

#### 4.2.2 Activation of Signaling Pathways

Building on the multifaceted influences of genetic mutations and extrinsic modulators, signaling pathways have emerged as pivotal intracellular hubs that transduce these disparate signals into coordinated lactylation responses, thereby amplifying metabolic reprogramming and tumor adaptation. By hijacking glycolytic cascades and epigenetic writers, these pathways not only elevate the production of lactate substrates, but also imprint lactyl marks on histones and nonhistone proteins, fostering an immunosuppressive niche that sustains oncogenesis.

In the glioblastoma TME, glioma‐derived factors propel MDMs toward a pro‐tumorigenic phenotype, where endoplasmic reticulum stress activates the PERK‐ATF4 axis to upregulate glucose transporter 1 (GLUT1) expression [159, 160]. This surge in glucose uptake fuels rampant glycolysis and lactate accrual, culminating in histone lactylation that enhances the expression of immunosuppressive genes, such as *IL‐10*, thereby blunting cytotoxic T cell infiltration and therapeutic efficacy [114, 161]. The transcription factor ZEB1, a master regulator of epithelial‐mesenchymal transition (EMT), transcriptionally reprograms glycolytic enzymes, including HK2 and PKM2, to entrench the Warburg effect in tumor cells. The resulting lactate overflow mediates global histone lactylation, augmenting chromatin accessibility at neuroectodermal loci to drive cellular plasticity, stemness, and metastatic dissemination [162, 163]. Extending this paradigm to gastric cancer (GC), Zhang et al. pioneered a predictive signature integrating hypoxia‐glycolysis‐lactylation‐related genes (HGLRGs) such as *LDHA* and *HIF1A*, which stratifies patients by risk and reveals the centrality of lactylation in hypoxic niches where glycolytic rewiring intersects with carcinogenic cascades. This model not only predicts survival, but also highlights therapeutic windows, as HGLRG dysregulation correlates with immune evasion and chemoresistance [164]. In liver metastases of CRC, G protein‐coupled receptor 37 (GPR37) exemplifies pathway precision by activating the Hippo‐YAP/TAZ effector arm. Specifically, GPR37 induces the transcription of *LDHA*, escalating glycolysis and H3K18la at chemokine promoters (CXCL1 and CXCL5). This lactylation event paradoxically boosts neutrophil chemoattraction while impairing antitumor functionality, creating a pro‐tumorigenic TME that accelerates metastatic seeding [165, 166, 167, 168]. STAT5 exhibits constitutive activation in AML cohorts, where it directly binds to and transactivates glycolytic gene promoters (including HK1, PFKP, and PDHA), thereby enhancing glycolytic flux in leukemic cells. This metabolic reprogramming increases intracellular lactate accumulation, which subsequently drives the nuclear translocation of E3BP and catalyzes extensive histone lactylation. Critically, lactylation of histone marks at the PD‐L1 locus and epigenetically remodels the chromatin landscape, ultimately promoting PD‐L1 transcriptional upregulation and establishing an immunosuppressive microenvironment in AML [95]. In summary, signaling pathways such as PERK‐ATF4, Hippo, and STAT5 converge on the association between glycolysis and lactylation to maintain tumor dominance, revealing reciprocity: lactate not only accumulates but also actively reprograms the epigenome to echo pathway dictates. This pattern positions lactylation as a metabolic‐epigenetic connection.

#### 4.2.3 Microbial Communities

The interplay between the gut microbiome and cancer is largely mediated by the persistent activation of the host immune system by microbial communities, which can either bolster antitumor defenses or inadvertently fuel neoplastic progression [169, 170, 171, 172]. Gut‐derived bacteria promote antitumor immune responses through multifaceted mechanisms, including the priming of T cell responses against bacterial antigens that exhibit molecular mimicry with tumor neoantigens, thereby eliciting cross‐reactive cytotoxicity; the engagement of pattern recognition receptors such as Toll‐like receptors and NOD‐like receptors, which orchestrate pro‐inflammatory or immunoregulatory cascades; and the exertion of systemic effects via microbial metabolites that modulate distant immune checkpoints and epigenetic landscapes [173, 174, 175]. Building on our prior emphasis on the pivotal role of lactate in driving protein lactylation, a dynamic PTM that covalently attaches lactyl groups to lysine residues, thereby linking glycolytic metabolism to epigenetic reprogramming, emerging evidence positions microbial metabolism as a critical, yet underexplored, source of lactate within the host ecosystem. We hypothesized that gut microorganisms regulate protein lactylation by modulating local and systemic lactate pools, thereby imprinting metabolic‐epigenetic signatures that propagate oncogenic signals. This conjecture aligns with the observation that dysbiotic microbiomes in patients with cancer amplify lactate production, fostering an acidic TME that is conducive to tumor adaptation.

Lactic acid bacteria (LAB), a phylogenetically diverse group known for fermenting carbohydrates into copious amounts of lactic acid under anaerobic conditions, exemplify this paradigm [176, 177, 178]. A consistent increase in the abundance of LAB, including those of the genera *Streptococcus*, *Lactobacillus*, and *Bifidobacterium*, has been observed in patients with GC. It has been confirmed that LAB affects GC through various mechanisms, such as the supply of exogenous lactate—a fuel for inflammation, angiogenesis, metastasis, EMT, and immunity evasion in cancer cells [178, 179]. Intriguingly, this microbial lactate influx may extend beyond direct fueling to epigenetic orchestration; by elevating substrate availability, LAB can amplify histone lactylation at pro‐oncogenic loci (e.g., H3K18la), thereby upregulating genes involved in EMT and metastasis while remodeling the TME toward immunosuppression. Despite these associations, empirical investigations into the microbial modulation of lactylation remain nascent, with few studies elucidating the precise pathways linking gut dysbiosis, lactate flux, and lactylome alterations in GC. This knowledge gap represents a frontier for translational research: prospective cohort studies integrating multi‐omics (metagenomics, metabolomics, and lactylome profiling) could elucidate LAB‐specific contributions to lactylation‐driven phenotypes, while gnotobiotic mouse models colonized with GC‐associated LAB strains might validate causality and therapeutic vulnerabilities. Targeting this axis via probiotics engineered to deplete lactate, LDHA inhibitors, or lactylation antagonists holds transformative potential, not only for enhancing immunotherapy efficacy in microbiome‐refractory GC but also for pioneering microbiome‐centric precision oncology. Ultimately, unraveling the microbial‐lactylation nexus could redefine cancer as a “holobiont” disease, where therapeutic success hinges on harmonizing host‐microbe metabolic dialogues to restore immune equilibrium and thwart metastatic cascades.

#### 4.2.4 Other Factors

Viruses, drugs, and environmental factors can also influence the metabolic microenvironment, induce protein lactylation, and ultimately affect cellular state and survival. The oncogenic DNA virus Kaposi's sarcoma‐associated herpesvirus (KSHV) regulates N‐acetyltransferase 10 (NAT10) and α‐tubulin acetyltransferase 1 (ATAT1), leading to the lactylation of NAT10. The latter results in the modification of tRNA^Ser‐CGA‐1‐1^ac^4^C, which ultimately promotes KSHV reactivation [180, 181, 182]. Human papillomavirus (HPV‐16) provides another compelling case in which the viral E6 protein inhibits the lactylation of G6PD at K45, activating the pentose phosphate pathway and fueling cervical cancer cell proliferation, a mechanism that highlights the role of lactylation as a metabolic checkpoint in virus‐driven carcinogenesis [183]. Oncolytic viruses (OV) can specifically replicate and cause apoptosis in cancer cells while protecting normal tissues from destruction [184]. Viruses often hijack host cell metabolic pathways for replication, and OVs are closely associated with a variety of specific tumor metabolic pathways, including glycolysis, pyruvate metabolism, the TCA cycle, and oxidative phosphorylation [185, 186]. However, it remains unknown whether OVs cause lactate metabolic reprogramming in vivo and the impact of this process on tumors; exploitation of this property could increase the clinical efficacy of OVs.

On the pharmacological front, drugs like TMZ inhibit histone lactylation through PPARα activation, sensitizing glioblastoma cells to therapy by altering lipid metabolism pathways. Moreover, statins, such as simvastatin, target the H3K18la/ACAT2 axis and enhance anti‐PD‐1 therapy in prostate cancer, revealing lactylation as a therapeutic vulnerability in lipid‐related cancers [187]. Acetaminophen (APAP) is widely used as an antipyretic and analgesic agent. In APAP‐induced hepatic injury, APAP therapy has been reported to reduce the expression of SIRT1/PGC‐1α/LDHB, increase the level of liver mitochondrial protein lactylation, and mediate liver pathology.

In PM2.5‐associated pulmonary fibrosis, researchers have found that PM2.5 induced glycolysis to produce lactylation, which promotes histone lactylation. Increased histone lactylation promotes profibrotic gene expression [188, 189, 190]. The pro‐fibrosis cytokines secreted by macrophages in the lung epithelium have been shown to induce EMT by activating TGF‐β/Smad2/3 and VEGFA/ERK. Furthermore, in mice, pretreatment with LDHA inhibitors (GNE‐140) effectively alleviated pulmonary inflammation and fibrosis induced by the inhibition of glycolysis and the subsequent modification of histone lactylation caused by PM2.5 exposure [191, 192]. Generally, factors that impact tumor metabolic reprogramming may regulate the tumorigenesis process by lactylation modification.

## 5 Clinical Trials of Lactylation‐Related Drugs

No specific human clinical trials have directly focused on “lactylation” as an intervention; however, lactylation is a rapidly evolving research area with potential for future therapeutic development, particularly in oncology. As shown in Table 2, the mechanism of current targeted drugs related to lactylation involve inhibiting lactate production (e.g., via LDHA), transport (e.g., via MCT1), or the “writer” enzyme p300. This review focuses on drugs in the clinical and preclinical stages of cancer therapy, drawing on recent studies up to the year 2025.

**TABLE 2:** Clinical trials of lactylation‐related drugs.

Drug Name	Target	Cancer type	Clinical trial number
2‐deoxy‐D‐glucose	Hexokinase	Solid tumors	NCT00096707
Dichloroacetate	Pyruvate dehydrogenase kinase	Previously treated metastatic breast cancer or NSCLC	NCT01029925
AZD3965	MCT1	Advanced solid tumors	NCT01791595
PRI‐724	CBP/β‐catenin	Advanced myeloid malignancies; pancreatic adenocarcinoma	NCT01606579; NCT01764477; NCT01302405
CCS‐1477	CBP/p300	Advanced solid tumors; hematological malignancies (NSCLC, mCRPC, metastatic breast cancer, non‐Hodgkin lymphoma, multiple myeloma, AML, high‐risk MDS)	NCT03568656; NCT04068597
FT‐7051	CBP/p300	Metastatic castration‐resistant prostate cancer	NCT04575766
NEO2734	BET/CBP/p300	Castration‐resistant prostate cancer; NUT midline carcinoma; Advanced solid tumors	NCT05488548

*Note*: Data sources—https://clinicaltrials.gov.

AZD3965, a selective MCT1 inhibitor, blocks lactate efflux, causing intracellular accumulation and disruption of lactylation‐dependent metabolic reprogramming. In a phase I dose‐escalation study (NCT01791595), AZD3965 demonstrated tolerability in advanced solid tumors, with a maximum tolerated dose of 20 mg daily via the oral route, showing target engagement by reducing lactate export in lymphomas and colon cancers. Preclinical data support the role of AZD3965 in enhancing immune infiltration by reshaping TME [193, 194].

P300 inhibitors, which reduce histone lactylation (e.g., H3K18la), target the acetyltransferase acting as a lactylation writer. Compounds like C646 have been shown to decrease H3K18la levels, reversing the activation of NF‐κB signaling and release of pro‐inflammatory cytokines in T cell acute lymphoblastic leukemia [195]. Meanwhile, A‐485 suppresses protein lactylation, alleviating neuronal death and glial activation in prostate cancer. Ongoing clinical trials have evaluated p300 inhibitors in multiple cancers with evidence of improved outcomes. These agents show promise for overcoming drug resistance, particularly in lactylation‐driven tumors [196, 197, 198, 199].

The development of drugs that directly target specific protein lactylation sites remains challenging. The main difficulty lies in the fact that lactylation is a widespread and dynamic PTM with high site specificity. Developing small‐molecule inhibitors that can accurately recognize specific lactylation sites and effectively regulate their function is extremely difficult. Therefore, no drugs that directly target protein lactylation have entered clinical research, and current strategies (such as the MCT1 inhibitor AZD3965) only indirectly affect the overall lactylation state by modulating intracellular lactate levels.

Looking ahead, the development of lactylation‐targeting drugs may progress in several directions. First, utilizing technologies like PROTACs (proteolysis‐targeting chimeras) to develop molecular glues or degraders that target lactylation “reader” or “writer” proteins, thereby interrupting specific lactylation signaling pathways more precisely. Second, high‐affinity monoclonal antibodies or peptide‐based substances for key proteins driven by lactylation in specific cancers with clear “oncogenic” should be developed to block their functions after lactylation. Ultimately, as the biological functions of lactylation are further elucidated, and technologies, such as CRISPR screening, are applied, more druggable targets may be discovered, paving the way for direct lactylation‐modulating drugs to enter clinical stages of development.

## 6 Visualization and Diagnosis of Lactate and Lactylation Modifications

In recent years, advancements in visualization and diagnostic technologies have accelerated, focusing on noninvasive imaging, specific biomarkers, and high‐throughput detection methods. These developments have facilitated early diagnosis, treatment monitoring, and mechanistic insights in the development of various diseases, especially cancer [200]. In the realm of lactate accumulation visualization and diagnosis, metabolic imaging techniques have become pivotal for the real‐time monitoring of glycolytic pathways, such as the conversion of pyruvate to lactate. The key challenges in detecting glycolytic pathway products include low lactate concentrations and high tissue heterogeneity; however, recent methods have shown enhanced sensitivity and spatial resolution. For instance, hyperpolarized ^13^C lactate magnetic resonance imaging (MRI) utilizes [1–^13^C] lactate tracers to amplify signals by several orders of magnitude, enabling the visualization of downstream metabolites, such as lactate and alanine. This test is of great significance for gliomas as well as renal, pancreatic, and cervical cancers. [201]. Clinical progress in 2023 demonstrated its ability to quantify the conversion rate of pyruvate to lactate in cancers, aiding in the assessment of tumor metabolic reprogramming and therapeutic responses. It also demonstrated the feasibility of identifying metabolically active lesions with a risk of postoperative biochemical recurrence in patients with intermediate‐risk prostate cancer [202]. Hyperpolarized ^13^C lactate MRI is particularly suitable for treating brain and prostate cancers.

Deuterium metabolic imaging, which is based on ^2^H‐MRS/MRSI, employs [6,6'‐^2^H2] glucose tracers to indirectly detect lactate via hydrogen‐deuterium exchange. Optimizations in 2024, including the Ernst‐angle sequences, improved the signal‐to‐noise ratios, achieving spatial resolutions of 3.3 mL at a 3‐T field strength. This imaging modality has been applied to observe the Warburg effect through elevated lactate/glutamine (Glx) ratios in tumors, strokes, and in cardiac metabolism. Moreover, this detection technology has been studied in various solid cancers, such as lymphoma, breast cancer, CRC, brain cancer, pancreatic ductal adenocarcinoma, and renal carcinoma [203].

Functional magnetic resonance spectroscopy combined with J‐editing has been shown to detect lactate signals in brain tissues. A 2023 application in Alzheimer's disease linked elevated lactate peaks to cognitive decline, and integration with dynamic ^13^C‐MRS allowed the imaging of glucose‐lactate pathways [204]. Positron emission tomography using L‐3‐^11^C‐lactate tracers quantifies lactate metabolism in the myocardium or glioblastoma, with advancements involving the combination of ^18^F‐FDG to evaluate increased lactate utilization in metastases. Offering high‐resolution images using these modalities (approximately 4 mm) is limited by radiation exposure [205, 206].

Regarding lactylation modifications, single‐cell fluorescence imaging using YnLac chemical probes for the in vivo visualization of lactylated proteins has elucidated gene expression heterogeneity [207]. In conclusion, these emerging technologies emphasize the shift toward integrating metabolic imaging with epigenetic profiling. However, the visualization of lactylation modification mainly relies on nascent tools such as YnLac probes and emerging genetic code expansions to gain site‐specific insights into living cells. This finding highlights a critical gap. Bridging this gap through multi‐omics innovations could bring about transformative trends in the real‐time, noninvasive monitoring of disease‐associated PTMs, ultimately promoting targeted interventions in cancer.

## 7 Conclusion and Prospects

Current developments in the burgeoning field of protein lactylation, a lactate‐derived PTM, highlight its pivotal role in bridging metabolic reprogramming and tumor biology. As detailed throughout this review, lactylation has emerged as a dynamic regulator that not only facilitates tumor progression through epigenetic and non‐epigenetic mechanisms but also profoundly influences therapeutic responses across chemotherapy, targeted therapies, and immunotherapies. Originating from the Warburg effect‐driven accumulation of lactate, lactylation modifies histones and nonhistone proteins via “writers,” “erasers,” and “readers,” thereby altering gene expression, protein stability, and cellular phenotypes. The involvement of this modification in DNA repair enhancement, autophagy promotion, immune evasion, and metabolic adaptation underscores its significance in multifaceted cancer resistance mechanisms. Lactylation holds immense promise for advancing tumor therapy, encompassing four key dimensions: (i) molecular markers for therapeutic response prediction, (ii) unveiling novel mechanisms of drug resistance, (iii) identifying new therapeutic targets, and (iv) providing insights into drug interactions and combination strategies.

First, lactylation‐related molecules serve as robust biomarkers for forecasting and assessing therapeutic efficacy, thereby offering a scientific foundation for personalized treatment regimens. For example, prognostic models integrating lactate‐associated genes, such as those derived from 108 algorithmic combinations in breast cancer, stratify patients into high‐ and low‐risk groups, with low‐risk cohorts exhibiting enhanced immune checkpoint expression and infiltration, suggesting better immunotherapy outcomes [122]. This example highlights the potential of lactylation as a noninvasive biomarker, which can be detected via liquid biopsies or tissue profiling, to guide patient stratification and monitor treatment dynamics in real time.

Second, lactylation research has unraveled innovative mechanisms underlying drug resistance, linking metabolic fluxes to epigenetic and signaling aberrations, revealing lactylation as a convergence point for metabolic‐epigenetic crosstalk. This may explain why tumors adapt to diverse therapies, emphasizing the need for deeper mechanistic studies to refine current therapeutic resistance models.

Third, lactylation represents a novel therapeutic target, shifting the focus from irreversible genetic alterations to modifiable metabolic and epigenetic nodes. Inhibitors of lactate production, such as LDHA blockers (e.g., oxamate and stiripentol), have shown preclinical promise in reversing resistance. Specifically, stiripentol synergizes with cisplatin or irradiation by curbing lactylation‐driven DNA damage repair [12], while oxamate enhances CAR‐T cell therapy in glioblastoma by reducing ectonucleotidase expression and Treg infiltration [93]. Targeting “writers” like p300 with compounds such as C646 or A‐485 diminishes H3K18la, alleviating pro‐inflammatory responses and neuronal damage in lactylation‐dependent contexts [196, 197]. Moreover, MCT1 inhibitors like AZD3965 have been shown in phase I trials to disrupt lactate efflux, indirectly attenuate lactylation, and reshape the TME to boost immune responses [193]. Finally, emerging strategies, including PROTACs as lactylation regulators or antibodies against lactylated proteins (e.g., IGF‐1R in multiple myeloma [66]), could enable the development of precise interventions. Furthermore, dual‐targeting mechanisms, such as combining lactylation inhibitors with PDGFRβ blockers in ccRCC, exploits positive feedback loops to enhance treatment efficacy [155]. As clinical trials progress (e.g., CCS‐1477 for p300 in solid tumors in NCT03568656), lactylation modulation may be integrated into precision oncology, particularly in metabolically aggressive cancers.

Fourth, lactylation offers new clues to drug interactions, fostering the development of synergistic combinations that can overcome the limitations of monotherapy. For example, HDAC inhibitors such as vorinostat enhance ferroptosis in CRC by reducing HDAC1K412la and destabilizing *FSP1* mRNA [70], suggesting potential synergy with ferroptosis inducers. In melanomas, LSD1 inhibitors reverse BRAFi/MEKi resistance by blocking lactate‐induced FosL1 stabilization [71]. Repurposed drugs, such as stavudine for TMZ sensitization in glioblastoma [44] or isosafrole for immunotherapy resensitization in brain tumors [116], exemplify how lactylation inhibition amplifies the therapeutic efficacy of existing agents. Extrinsic factors, such as microbiome modulation (e.g., targeting LAB in GC [178, 179]) or environmental interventions (e.g., PM2.5 exposure mitigation via LDHA inhibitors), could indirectly influence lactylation‐drug dynamics. Positive results with viral influences, such as KSHV‐induced NAT10 lactylation [180, 181], hinted at combining antivirals with lactylation modulators. These interactions underscore the role of lactylation in polypharmacology, where metabolic interventions can preempt treatment resistance and enhance the durability of the therapeutic response.

In summary, mounting evidence suggests that lactylation is a critical bridge between cellular metabolism and phenotypic reprogramming, profoundly modulating tumor therapeutic responses. By integrating insights from metabolic, epigenetic, and immunological perspectives, future research should prioritize comprehensive lactylome mapping, isoform‐specific analyses (e.g., Kla vs. Kd‐la [8]), and multi‐omics integration to accelerate biomarker validation and drug development.

Nonetheless, challenges still remain, including site‐specific targeting and clinical translation, technical limitations in the precise detection and quantification of lactylation sites using methods such as mass spectrometry, which require optimization of clinical samples, and the lack of specific antibodies to detect certain modified sites. Biological complexities arise from uncertainties regarding the exact mechanisms of lactylation, such as competition with other PTMs, acetylation at shared lysine residues, the absence of identified specific delactylases, and their dual roles in driving or reflecting metabolic changes. Translational hurdles include reliance on preclinical models without sufficient clinical cohort validation, metabolic heterogeneity across tumor types leading to potential off‐target effects, and overlapping pathways that complicate the attribution of treatment resistance solely to lactylation. Therapeutic challenges include redundancy in resistance mechanisms, necessitating combination strategies, and the need for more precise inhibitors to avoid broader metabolic disruptions. Despite these challenges, the reversible nature of lactylation presents a transformative opportunity to overcome treatment resistance and advance personalized cancer care. Ultimately, lactylation has the potential to reshape therapeutic strategies, paving the way for a metabolism‐focused approach in oncology that enhances patient outcomes.

## Author Contributions

Conceptualization: Hui Zhou and Ling Xiao. Article writing and editing: Qianying Ouyang and Qianyu Hu. Figure organization: Qianying Ouyang and Qianyu Hu. Compilation of literature: Caiqin Wang, Yizi He, Ruolan Zeng, Yajun Li, Chang Su, Guige Lu, and Xueting Zhu. Supervision: Hui Zhou and Ling Xiao. All authors read and approved the final manuscript.

## Ethics Statement

The authors have nothing to report.

## Conflicts of Interest

The authors declare no conflicts of interest.

## Data Availability

The authors have nothing to report.
